# Perioperative tislelizumab plus chemotherapy for locally advanced resectable thoracic esophageal squamous cell carcinoma trial: a prospective single-arm, phase II study (PILOT trial)

**DOI:** 10.1186/s12885-023-11747-9

**Published:** 2023-12-15

**Authors:** Chengzhi Ding, Yijun Guo, Yaning zhou, Yi He, Chunji Chen, Ming Zhang, Xufeng Guo

**Affiliations:** 1 Department of Thoracic Surgery, Shanghai Chest Hospital, Shanghai Jiao Tong University School of Medicine, Shanghai, 200030 China; 2 Department of Thoracic Surgery, Henan Provincial People’s Hospital, Zhengzhou University People’s Hospital, Zhengzhou, 450003 China; 3 Department of Integrated Traditional Chinese and Western Medicine, Shanghai Chest Hospital, Shanghai Jiao Tong University School of Medicine, Shanghai, 200030 China

**Keywords:** Esophageal squamous cell carcinoma (ESCC), Tislelizumab, Esophagectomy, Neoadjuvant, Adjuvant, Immunotherapy

## Abstract

**Background:**

The promising therapeutic outcomes of neoadjuvant immunotherapy combined with chemotherapy in the treatment of locally advanced esophageal squamous cell carcinoma (ESCC) have been confirmed by several phase II clinical trials and have been widely demonstrated in clinical work. Theoretically, postoperative adjuvant immunotherapy may further improve the therapeutic effect, but there is still lack of evidence. The aim of this study was to analyse the safety and efficacy of perioperative immunotherapy (tislelizumab) in locally advanced resectable thoracic ESCC (PILOT trial).

**Methods:**

Seventy-three eligible patients with pathologically confirmed thoracic ESCC of clinical T1b-3N1-3M0 or T3N0M0 stage were allocated to receive neoadjuvant immunotherapy (tislelizumab 200 mg d1, q3w × 2 cycles) plus chemotherapy (nad-paclitaxel 260 mg/m^2^ d1 + carboplatin AUC = 5 d1, q3w × 2 cycles) treatment. Patients with pathologic complete response (pCR) after esophagectomy received adjuvant tislelizumab (200 mg every 3 weeks for up to one year), and patients with non-pCR were assigned adjuvant tislelizumab plus chemotherapy for two cycles and then maintenance tislelizumab (200 mg every 3 weeks for up to 15 cycles). The primary endpoint of this study is 2-year disease-free survival (DFS) in non-pCR patients. The secondary endpoints include pCR rate, major pathological response rate, 2-year DFS in pCR patients, R0 resection rate, adverse events, and overall survival.

**Discussion:**

This protocol was reviewed and approved by the Ethics Committee of Shanghai Chest Hospital (IS23059). This is the first prospective clinical trial to investigate the safety and efficacy of perioperative immunotherapy for locally advanced resectable thoracic ESCC. We hypothesize that perioperative immunotherapy could be a promising therapeutic strategy that can provide better 2-year DFS in non-pCR patients.

**Trial registration:**

ClinicalTrial.gov: NCT0605633.

## Background

Esophageal cancer ranks as the sixth most common cause of cancer death globally [1]. Nearly half of new cases worldwide are diagnosed in China every year, where the histological type is mainly esophageal squamous cell carcinoma (ESCC) [2]. Most ESCC cases are already in a locally advanced stage upon presentation, and surgery alone is far from sufficient as a result of the high recurrence or metastasis rates [3].

The CROSS study and NEOCRTEC5010 study confirmed that neoadjuvant chemoradiotherapy (nCRT) can significantly improve the long-term survival of ESCC patients after surgery [4–6]. However, the 5 to 10-year overall failure rate after nCRT followed by esophagectomy is as high as 34.6% ~ 48.0% [6, 7]. In the CROSS study, the majority of patients who received nCRT had a recurrence and metastasis rate of 34.7% during a median follow-up of 2 years after surgery, with a distant metastasis rate of 22% [7]. Long-term follow-up results of the NEOCRTEC5010 study showed that 33.0% of patients who had received nCRT still had recurrence and metastasis during the 3-year follow-up after surgery, and distant metastasis (21.4%) was more common [8]. All this evidences suggests that more efficient systemic treatment options are required to further improve efficacy in locally advanced resectable ESCC.

In recent years, neoadjuvant immunotherapy combined with chemotherapy (nICT) has been the most promising research frontier for multidisciplinary treatment of locally advanced ESCC [9–12]. A multicentre real-world study also confirmed the safety and efficacy of nICT in ESCC [13]. Based on this, a phase III clinical study of nICT versus nCRT in the treatment of locally advanced ESCC is being conducted to further evaluate whether nICT can be a better strategy in the future [14]. The CheckMate 577 trial showed that nivolumab could significantly prolong disease-free survival (DFS) in non-pathologic complete response (pCR) patients after nCRT [15]. However, whether adjuvant immunotherapy provides additional benefits for patients receiving nICT remains an unanswered question.

Hence, we designed this prospective single-arm, phase II trial (PILOT trial) to assess the safety and efficacy of perioperative immunotherapy in locally advanced resectable ESCC and answer the questions that CheckMate 577 did not cover.

## Methods/design

### Study design

**Fig. 1:**
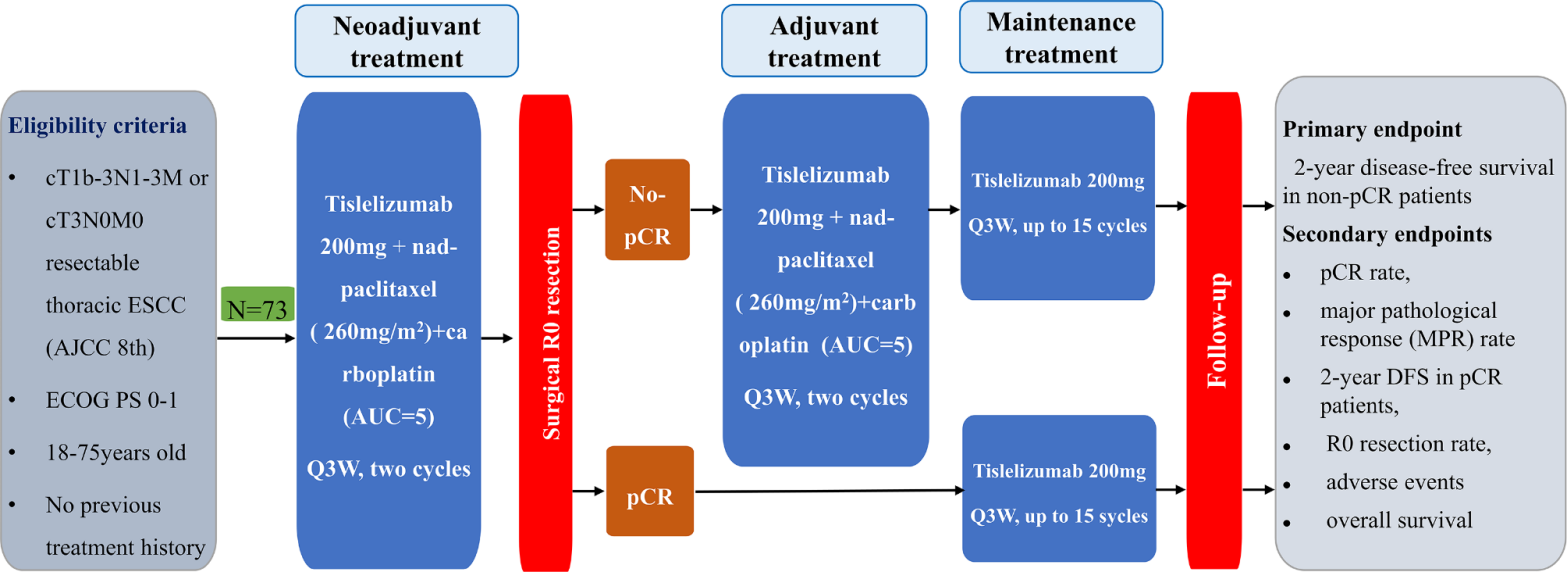
Flow chart of the PILOT trial

The PILOT trial was a prospective, single-arm phase II study of perioperative immunotherapy for locally advanced resectable thoracic ESCC. The primary endpoint was 2-year DFS in non-pCR patients, which was defined as the time from the first day of surgery to the first recurrence or death from any cause. The secondary endpoints were pCR rate, major pathological response (MPR) rate, 2-year DFS in pCR patients, R0 resection rate, adverse events, and overall survival (OS). pCR was defined as the complete response of both the primary tumour and lymph nodes in the resected specimen. Recurrence was defined as the appearance of one or more new lesions (confirmed by imaging or cytologic or pathological assessment, local, regional, or distant from the primary resection site) as assessed by the investigator. The trial flow chart is presented in Fig. 1. The study started on January 1, 2024, and the planned closing date is December 31, 2026.

### Study procedures

Eligible patients agreed to written informed consent before enrollment and were assigned to receive adjuvant immunotherapy following esophagectomy after nICT. All patients received neoadjuvant immunotherapy (tislelizumab 200 mg d1, q3w× 2 cycles) and chemotherapy (nad-paclitaxel 260 mg/m^2^ d1 + carboplatin AUC = 5 d1, q3w × 2 cycles) treatment. The efficacy was evaluated according to RECIST 1.1. Patients with clinical complete response (cCR), clinical partial response (cPR), and clinical stable disease (cSD) underwent surgery after 4 to 6 weeks. If patients were evaluated for clinical progressive disease (cPD), they received a new treatment regimen after a multidisciplinary treatment discussion. After surgery, patients with pCR always underwent adjuvant immunotherapy (tislelizumab 200 mg q3w) up to one year, and patients with non-pCR were assigned to receive adjuvant immunotherapy (tislelizumab 200 mg d1, q3w × 2 cycles) plus chemotherapy (nad-paclitaxel 260 mg/m^2^ d1 + carboplatin AUC = 5 d1, q3w × 2 cycles) and then maintenance tislelizumab (200 mg every 3 weeks for up to 15 cycles). Those patients who did not undergo R0 resection received adjuvant chemoradiotherapy.

Treatment could be interrupted or postponed if any serious adverse effects occured, and treatment could be resumed until the established standards for resumption of treatment were met. The safety-related endpoints included neoadjuvant and adjuvant treatment-related adverse events (TRAEs), immune-related adverse events (irAEs), resection rate, and surgical complications. TRAEs and irAEs were evaluated according to the National Cancer Institute Common Terminology Criteria for Adverse Events, version 5.0 (CTCAE5.0) [16]. Serious adverse events (SAEs) were defined as grade 3 to 5 TRAEs. If SAEs occur during the trial, the investigator was expected to immediately take appropriate protective measures or discontinue the treatment, report to the principal investigator within 24 h, and fill out and sign the SAE report form. The resection rate refers to the ratio of patients who underwent surgical resection to the intention-to-treat population. Surgical complications within 30 days after operation were coded using the Clavien-Dindo classification. All investigators were requried to comply with the standard operating procedures for the management of study drugs.

The enrolled patients received thoracoscopy combined with laparoscopy -assisted esophagectomy using the McKeown procedure, including thoraco-abdominal two-field lymphadenectomy.The lymph node stations dissected were as follows [17]: the left recurrent laryngeal nerve nodes, right recurrent laryngeal nerve nodes, infra-aortic arch nodes, peri-esophageal nodes of the upper, middle and lower thoracic portion, infracarinal nodes, posterior mediastinal nodes, paracardiac nodes, lesser curvature nodes, left gastric nodes, common hepatic nodes, splenic nodes and celiac nodes. Dissection of the bilateral recurrent laryngeal nerve nodes was mandatory. A small incision was made under the xiphoid process to make a tubular stomach, and esophagogastric mechanical anastomosis was performed through a left vertical neck incision along the anterior border of the sternocleidomastoid muscle.

### Eligibility criteria

#### Inclusion criteria

1. The patient volunteers agreed to participate in the study, signed a consent form, exhibited good compliance, observed the follow-up procedures, and were willing and able to follow the protocol during the study.
2. Histologically-confirmed squamous cell carcinoma; tumors of the esophagus are located in the thoracic cavity.
3. The patients had not received systemic or local treatment for esophageal cancer.
4. Pretreatment staging was cT1b-3N1-3M0 or T3N0M0, American Joint Committee on Cancer (AJCC)/Union for International Cancer Control (UICC) 8th edition.
5. Male or female, aged ≥ 18 and ≤ 75 years.
6. The Eastern Cooperative Oncology Group (ECOG) performance status (PS) score is 0–1.
7. R0 resection is expected.
8. Adequate cardiac function: all patients were expected to undergo electrocardiogram (ECG), and those with a cardiac history or ECG abnormality were expected to undergo echocardiography with left ventricular ejection fraction > 50%.
9. Adequate respiratory function with forced expiratory volume in 1 s (FEV1) ≥ 1.2 L, FEV1% ≥ 50%, and lung diffusing capacity for carbon monoxide (DLCO) ≥ 50% shown in pulmonary function tests.
10. Adequate bone marrow function (white blood cells > 4 × 10^9^/L, neutrophil > 1.5 × 10^9^/L, haemoglobin > 90 g/L, platelets > 100 × 10^9^/L);aspartate aminotransferase (AST), alanine aminotransferase (ALT) ≤ 3× upper level of normal (ULN).
11. Adequate liver function (total bilirubin < 1.5× ULN, AST and ALT < 2.5× ULN).
12. Adequate renal function (glomerular filtration rate (GFR) > 60 mL/min; serum creatinine (SCr) ≤ 120 µmol/L].
13. Fertile female subjects were e required to have a negative serum or urine pregnancy test no later than 72 h before starting the study drug administration, and to use effective contraception (such as an IUD, contraceptive pill, or condom) during the trial period and for at least 3 months after the last dose; male subjects whose partners were women of reproductive age; were instructed to use effective contraception should be used during the trial period and within 3 months after the last dose.

#### Exclusion criteria

1. Unresectable factors, including those who are unresectable for tumor reasons or have surgical contraindications, or who refuse surgery.
2. Patients with supraclavicular lymph node metastasis.
3. Poor nutritional status, BMI < 18.5 kg/m2; patients could continue to be considered for enrollment if corrected with symptomatic nutritional support before enrollment and after assessment by the principal investigator.
4. Allergy to any drugs.
5. Have received or are receiving any of the following treatments; (a) any radiotherapy, chemotherapy, or other anti-neoplastic drugs directed at the tumour; (b) treatment with an immuno-suppressive drug or systemic hormone for immuno-suppression (at a dose of > 10 mg/ day of prednisone or equivalent) within 2 weeks before the first dose of the study drug; Inhaled or topical steroids and corticosteroid replacement at doses > 10 mg/ day of prednisone or equivalent were allowed in the absence of active autoimmune disease; (c) live attenuated vaccine within 4 weeks before the first dose of study drug; (d) major surgery or severe trauma within 4 weeks before the first dose of study drug.
6. Human immunodeficiency virus (HIV), hepatitis B virus (HBV) or hepatitis C virus (HCV) active infection, or known HIV seropositivity; including HBV or HCV surface antigen positive (RNA).
7. Uncontrolled cardiac symptoms or diseases, including but not limited to (1) heart failure above NYHA class II, (2) unstable angina, (3) myocardial infarction within 1 year, and (4) clinically important significant supraventricular or ventricular arrhythmias without or poorly controlled after the clinical intervention.
8. Severe infection (CTCAE > 2) occurring within 4 weeks before the first dose of the study drug, such as severe pneumonia requiring hospitalization, bacteremia, and infectious complications; patients using prophylactic antibiotics were excluded if there was active pulmonary inflammation on chest imaging at baseline, if there were signs and symptoms of infection within 14 days before the first dose of the study drug, or if treatment with oral or intravenous antibiotics was needed.
9. Participation in other drug clinical studies within 4 weeks before randomization.
10. Patients with interstitial pneumonia or interstitial lung disease, or previous history of interstitial pneumonia or interstitial lung disease requiring hormone therapy; or other subjects with pulmonary fibrosis, organized pneumonia (such as bronchiolitis obliterans), pneumoconiosis, drug-related pneumonia, idiopathic pneumonia that could interfere with the assessment and treatment of immune-related pulmonary toxicity; subjects with active pneumonia or severe lung function damage revealed by CT during screening; patients with active pulmonary tuberculosis.
11. Patients with any active autoimmune disease or history of autoimmune disease and possible recurrence [including but not limited to autoimmune hepatitis, interstitial pneumonia, uveitis, enteritis, hypophysitis, vasculitis, nephritis, hyperthyroidism, hypothyroidism (patients who can be controlled only by hormone replacement therapy can be enrolled); patients with skin diseases that do not require systemic treatment, such as leukoplakia, psoriasis, alopecia; patients with type I diabetes that can be controlled by insulin treatment; patients with a history of asthma, but those with childhood asthma not requiring intervention can be enrolled; asthma patients who needed bronchodilators for intervention; patients who had previously received an anti-PD-1, PD-L1 or any other antibody or drug specifically targeting T-cell costimulation or checkpoint pathways.
12. Other malignancies that had been diagnosed within 5 years before the first dose of a study drug were considered unless cancers with a low risk of metastasis or death (5-year survival rate, > 90%), such as adequately treated basal-cell or squamous-cell skin cancer or carcinoma in situ of the cervix, were considered.
13. Pregnant or lactating women.
14. The investigators determined that there were other factors that might lead to the forced discontinuation of the study, such as family or social factors, other serious medical conditions (including mental illness) requiring cotreatment, alcohol abuse, and substance abuse, as well as factors that could affect the safety or compliance of the subjects.

### Pathologic examination

The pathological report was consulted by two senior pathologists, including the depth of infiltration of the primary lesion, histological type, pathological status of the upper and posterior margins, and involvement of peripheral and lymph nodes. The tumor regression grade [18] and ypTNM staging was evaluated.

### Follow-up

Follow-up was to be first performed one month after surgery. From then on, follow-up visits was to be every 3 months in the first 2 years. The detailed examination items included standard laboratory tests (routine blood tests, tumor biomarkers), an enhanced CT scan of the thorax, and an ultrasound of the neck and abdomen.

If the patient had signs of recurrence (such as related clinical manifestations), additional tumor evaluations were to be performed during the treatment; possible reoperations and/or further cancer treatments were also to be documented. During the follow-up period without tumor recurrence, other cytotoxic agents were not allowed. Patient recurrence and survival was to be followed up until the patient’s death or the last date of the patients known survival.

### Statistics

The previous CheckMate 577 study suggested that the 2-year DFS was 58% for non-pCR patients with locally advanced ESCC who received nCRT followed by radical surgery. Assuming that after perioperative immunochemotherapy and maintenance immunotherapy, the 2-year DFS increases to 75%, the trial would required 55 eligible non-pCR patients with a level of significance of 90% (α = 0.2) and a power of 90% (β = 0.05). According to the previous retrospective data analysis of our center, the pCR rate of patients with the nICT regimen followed by surgery was 26.5%, referring to the published data of the multicenter real-world study of nICT in mainland China (pCR 25.8%), and 18 additional patients would be needed further. A total of 73 patients were needed to be enrolled in this study.

Two-year DFS in non-pCR patients, 2-year DFS in pCR patients and OS were calculated by the Kaplan-Meier method. The Cox proportional hazard model was used to evaluate the survival independent factors. Continuous variables are presented as the mean and standard deviation (SD). Categorical variables are presented as percentages and are compared using the chi-square test. The differences in proportions between the two groups were evaluated by Fisher’s exact test. The Wilcoxon test was used to compare the non-parametric datasets. All statistical analysis was to be calculated using SPSS (version 24.0, Chicago, IL, USA) statistical analysis software programming. *P* values less than 0.05 will be considered statistically significant.

## Discussion

The current standard treatment for locally advanced thoracic resectable ESCC is nCRT followed by esophagectomy [4, 19]. However, more than 50% of ESCC patients relapse within 2 years after nCRT, especially in non-pCR patients, and in a proportion of pCR patients [20]. According to the 10-year follow-up in the CROSS study, 27% of patients receiving nCRT developed distant metastasis, suggesting that nCRT alone is not sufficient to improve long-term survival [21].

The JCOG 1109 study showed that preoperative docetaxel chemotherapy, cisplatin, and 5-fluorouracil (DCF) regimen achieved higher survival benefits and significantly reduced postoperative recurrence and metastasis through stronger systemic treatment, especially postoperative distant metastasis (56.6%), which was significantly lower than that in the CF group (62.3%) and nCRT group (77.3%) [22]. The CMISG1701 trial demonstrated that nCRT achieved better local control and tumor regression in locally advanced ESCC than neoadjuvant chemotherapy (nCT), but the survival benefit was not significantly different [23]. Similarly, the CIMISG1701 trial also showed that nearly 40% of patients in the two groups still had recurrence and metastasis within 2 years after surgery (nCT vs. nCRT: 37.9% vs. 49.2%) with distant metastasis was occurring in 13.6% and 16.7%, respectively) [23]. Therefore, both nCT and nCRT still cannot be considered optimal treatment modes for locally advanced resectable ESCC, and it is necessary to find more efficient systemic treatment methods to reduce the risk of recurrence and metastasis.

Rapid advances in immunotherapy for ESCC have been achieved since numerous recent clinical trials reported positive results. ESCC has emerged as one of the most promising targets for immune checkpoint inhibitor treatment. Tislelizumab is a human IgG4 monoclonal antibody that binds to and blocks the PD-1 receptor expressed on activated immune cells, including T lymphocytes [24]. The efficacy and safety of tislelizumab in first-line and second-line treatment for metastatic ESCC have been confirmed in the RATIONALE-302 and RATIONALE-306 studies respectively [25, 26]. Recently, nICT was used to treat locally advanced resectable ESCC, and a pCR rate (18.8 ~ 50%) similar to that of nCRT was achieved [10–12]. In addition, TD-NICE and ETNT studies have shown that tislelizumab plus chemotherapy as neoadjuvant therapy can achieve a higher pCR rate, and cause less treatment-related adverse events [9, 27].

Furthermore, the CheckMate 577 trial showed that nivolumab could significantly prolong DFS in non-pCR patients after nCRT, and it is worth nothing that patients with ESCC benefited more than those with adenocarcinoma, with a 2-year DFS of 58% [15]. A real-world retrospective study included ninety locally advanced ESCC patients treated with nICT followed by surgery. Most patients were treated with adjuvant immunotherapy, and completed at least 1- year of follow-up. The 2-year DFS rate was 78.3% and the 2-year OS rate was 88.0% [28]. The two-year follow-up outcomes of the NICE1 study showed that among ESCC patients receiving nICT, after a median follow-up of 27.4 months, there was disease recurrence in 19 (37.3%) patients, with 9.8% locoregional recurrence, 17.6% distant metastasis, and 9.8% combined recurrence, and distant metastasis remained the predominant recurrence pattern [29]. Whether perioperative immunotherapy can further improve the long-term survival of patients with locally advanced ESCC has not been well evaluated thus far. Recently, the Neotorch trial [30] and Keynote 671 [31] have reported positive results of perioperative immunotherapy for resectable non-small cell lung cancer. In theory, adjuvant immunotherapy after nICT could help reduce postoperative recurrence and improve survival for ESCC patients.

Additionally, patients with ESCC who achieved pCR following nCRT (pCR rate: 44.9%) were followed up, and the overall recurrence rate within 5 years was reported to be 34.3% [32]. Moreover, the NEOCRTEC5010 study showed that 11 (13.9%) of 80 pCR patients had recurrence and metastasis during the 3-year follow-up after surgery [33], suggesting that pCR status does not indicate absolutely safety; whether it can also benefit from adjuvant immunotherapy needs to be further explored.

In summary, we designed this PILOT trial to assess whether perioperative immunotherapy would provide acceptable safety and better survival benefits for locally advanced thoracic ESCC patients. This present trial could help to answer the following questions: first, whether the combination of neoadjuvant tislelizumab and chemotherapy (TC regimen) exerts better tumor downstaging; second, whether adjuvant tislelizumab treatment offers additional survival benefit in non-pCR and pCR patients after nICT; and third, whether perioperative immunotherapy has an acceptable safety profile. It is hypothesized that perioperative immunotherapy could improve survival due to enhanced systemic treatment.

## Data Availability

The datasets used during the PILOT study are available from the corresponding author upon a reasonable request.

## Abbreviations

PD-1: Programmed cell death protein 1
AUC: Area Under the Curve
ESCC: Esophageal squamous cell carcinoma
pCR: Pathological complete response
non-pCR: Non-pathological complete response
cCR: Clinically complete response
cPR: Clinically partial response
cSD: Clinically stable disease
cPD: Clinically progressive disease
CRE: Clinical response evaluation
CRE: Clinical response evaluation
CTCAE: Common terminology criteria for adverse event
RECIST: Response evaluation criteria for solid tumors
nCRT: Neoadjuvant chemoradiotherapy
DFS: Disease-free survival (DFS)
OS: Sverall survival
PET/CT: Positron emission tomography/computed tomography
TNM: Tumor bode metastasis classification system
UICC: Union for International Cancer Control

## References

[1] Sung H, Ferlay J, Siegel RL, Laversanne M, Soerjomataram I, Jemal A, Bray F (2021). Global Cancer statistics 2020: GLOBOCAN estimates of incidence and Mortality Worldwide for 36 cancers in 185 countries. CA Cancer J Clin.

[2] Qiu H, Cao S, Xu R (2021). Cancer incidence, mortality, and burden in China: a time-trend analysis and comparison with the United States and United Kingdom based on the global epidemiological data released in 2020. Cancer Commun (Lond).

[3] van Hagen P, Hulshof MC, van Lanschot JJ, Steyerberg EW, van Berge Henegouwen MI, Wijnhoven BP, Richel DJ, Nieuwenhuijzen GA, Hospers GA (2012). Preoperative chemoradiotherapy for esophageal or junctional cancer. N Engl J Med.

[4] Shapiro J, van Lanschot JJB, Hulshof MCCM, van Hagen P, van Berge Henegouwen MI, Wijnhoven BPL, van Laarhoven HWM, Nieuwenhuijzen GAP, Hospers GAP (2015). Neoadjuvant chemoradiotherapy plus Surgery versus Surgery alone for oesophageal or junctional cancer (CROSS): long-term results of a randomized controlled trial. Lancet Oncol.

[5] Yang H, Liu H, Chen Y, Zhu C, Fang W, Yu Z, Mao W, Xiang J, Han Y, Chen Z (2018). Neoadjuvant chemoradiotherapy followed by Surgery versus Surgery alone for locally advanced squamous cell carcinoma of the esophagus (NEOCRTEC5010): a phase III multicenter, randomized, open-label clinical trial. J Clin Oncol.

[6] Yang H, Liu H, Chen Y, Zhu C, Fang W, Yu Z, Mao W, Xiang J, Han Y, Chen Z (2021). Long-term efficacy of Neoadjuvant Chemoradiotherapy Plus Surgery for the treatment of locally advanced esophageal squamous cell carcinoma: the NEOCRTEC5010 Randomized Clinical Trial. JAMA Surg.

[7] Oppedijk V, van der Gaast A, van Lanschot JJ, van Hagen P, van Os R, van Rij CM, van der Sangen MJ, Beukema JC, Rütten H, Spruit PH (2014). Patterns of recurrence after Surgery alone versus preoperative chemoradiotherapy and Surgery in the CROSS trials. J Clin Oncol.

[8] Liu S, Wen J, Yang H, Li Q, Chen Y, Zhu C, Fang W, Yu Z, Mao W, Xiang J (2020). Recurrence patterns after neoadjuvant chemoradiotherapy compared with Surgery alone in oesophageal squamous cell carcinoma: results from the multicenter phase III trial NEOCRTEC5010. Eur J Cancer.

[9] Yan X, Duan H, Ni Y, Zhou Y, Wang X, Qi H, Gong L, Liu H, Tian F, Lu Q (2022). Tislelizumab combined with chemotherapy as neoadjuvant therapy for surgically resectable Esophageal cancer: a prospective, single-arm, phase II study (TD-NICE). Int J Surg.

[10] Liu J, Li J, Lin W, Shao D, Depypere L, Zhang Z, Li Z, Cui F, Du Z, Zeng Y (2022). Neoadjuvant camrelizumab plus chemotherapy for resectable, locally advanced esophageal squamous cell carcinoma (NIC-ESCC2019): a multicenter, phase 2 study. Int J Cancer.

[11] Zhang Z, Hong ZN, Xie S, Lin W, Lin Y, Zhu J, Yang X, Lin Z, Lin J, Kang M (2021). Neoadjuvant sintilimab plus chemotherapy for locally advanced esophageal squamous cell carcinoma: a single-arm, single-center, phase 2 trial (ESONICT-1). Ann Transl Med.

[12] Liu J, Yang Y, Liu Z, Fu X, Cai X, Li H, Zhu L, Shen Y, Zhang H, Sun Y (2022). Multicenter, single-arm, phase II trial of camrelizumab and chemotherapy as neoadjuvant treatment for locally advanced esophageal squamous cell carcinoma. J Immunother Cancer.

[13] Yang Y, Tan L, Hu J, Li Y, Mao Y, Tian Z, Zhang B, Ma J, Li H, Chen C (2022). Esophageal Cancer Committee of Chinese Anti-cancer Association. Safety and efficacy of neoadjuvant treatment with immune checkpoint inhibitors in Esophageal cancer: real-world multicenter retrospective study in China. Dis Esophagus.

[14] Shang X, Zhang W, Zhao G, Liang F, Zhang C, Yue J, Duan X, Ma Z, Chen C, Pang Q (2022). Pembrolizumab Combined with Neoadjuvant Chemotherapy Versus Neoadjuvant Chemoradiotherapy followed by Surgery for locally advanced oesophageal squamous cell carcinoma: protocol for a Multicentre, prospective, Randomized-Controlled, phase III clinical study (Keystone-002). Front Oncol.

[15] Kelly RJ, Ajani JA, Kuzdzal J, Zander T, Van Cutsem E, Piessen G, Mendez G, Feliciano J, Motoyama S, Lièvre A (2021). Adjuvant nivolumab in Resected Esophageal or Gastroesophageal Junction Cancer. N Engl J Med.

[16] Dueck AC, Mendoza TR, Mitchell SA, Reeve BB, Castro KM, Rogak LJ, Atkinson TM, Bennett AV, Denicof AM, O’Mara AM (2015). Validity and reliability of the US National Cancer Institute’s patient-reported outcomes Version of the common terminology criteria for adverse events (PRO-CTCAE). JAMA Oncol.

[17] Japan Esophageal Society (2017). Japanese classification of Esophageal Cancer, 11th Edition: part II and III. Esophagus.

[18] Chirieac LR, Swisher SG, Ajani JA, Komaki RR, Correa AM, Morris JS, Roth JA, Rashid A, Hamilton SR, Wu TT (2005). Posttherapy pathologic stage predicts survival in patients with esophageal carcinoma receiving preoperative chemoradiation. Cancer.

[19] Ajani JA, D’Amico TA, Bentrem DJ, Cooke D, Corvera C, Das P, Enzinger PC, Enzler T, Farjah F, Gerdes H (2023). Esophageal and Esophagogastric Junction Cancers, Version 2.2023, NCCN Clinical Practice guidelines in Oncology. J Natl Compr Canc Netw.

[20] Leng X, He W, Yang H, Chen Y, Zhu C, Fang W, Yu Z, Mao W, Xiang J, Chen Z (2021). Prognostic impact of postoperative lymph node metastases after neoadjuvant chemoradiotherapy for locally advanced squamous cell carcinoma of esophagus: from the results of NEOCRTEC5010, a randomized multicenter study. Ann Surg.

[21] Eyck BM, van Lanschot JJB, Hulshof MCCM, van der Wilk BJ, Shapiro J, van Hagen P, van Berge Henegouwen MI, Wijnhoven BPL, van Laarhoven HWM, Nieuwenhuijzen GAP (2021). Ten-year outcome of Neoadjuvant Chemoradiotherapy Plus Surgery for Esophageal Cancer: the Randomized Controlled CROSS Trial. J Clin Oncol.

[22] Ken Kato Y, Ito H, Daiko S, Ozawa T, Ogata H, Hara T, Kojima T, Abe T, Bamba M, Watanabe (2022). A randomized controlled phase III trial comparing two chemotherapy regimen and chemoradiotherapy regimen as neoadjuvant treatment for locally advanced Esophageal cancer, JCOG1109 NExT study. J Clin Oncol.

[23] Tang H, Wang H, Fang Y, Zhu JY, Yin J, Shen YX, Zeng ZC, Jiang DX, Hou YY, Du M (2023). Neoadjuvant chemoradiotherapy versus neoadjuvant chemotherapy followed by minimally invasive esophagectomy for locally advanced esophageal squamous cell carcinoma: a prospective multicenter randomized clinical trial. Ann Oncol.

[24] Lee A, Keam SJ, Tislelizumab (2020). First Approval Drugs.

[25] Shen L, Kato K, Kim SB, Ajani JA, Zhao K, He Z, Yu X, Shu Y, Luo Q, Wang J (2022). Tislelizumab Versus Chemotherapy as Second-Line treatment for Advanced or metastatic esophageal squamous cell carcinoma (RATIONALE-302): a Randomized Phase III Study. J Clin Oncol.

[26] Xu J, Kato K, Raymond E, Hubner RA, Shu Y, Pan Y, Park SR, Ping L, Jiang Y, Zhang J (2023). Tislelizumab plus chemotherapy versus placebo plus chemotherapy as first-line treatment for advanced or metastatic oesophageal squamous cell carcinoma (RATIONALE-306): a global, randomised, placebo-controlled, phase 3 study. Lancet Oncol.

[27] He W, Wang C, Wu L, Wan G, Li B, Han Y, Li H, Leng X, Du K, Chen H (2022). Tislelizumab Plus Chemotherapy Sequential Neoadjuvant Therapy for Non-cCR patients after Neoadjuvant Chemoradiotherapy in locally advanced esophageal squamous cell carcinoma (ETNT): an exploratory study. Front Immunol.

[28] Lv H, Huang C, Li J, Zhang F, Gai C, Liu Z, Xu S, Wang M, Li Z, Tian Z (2022). The survival outcomes of neoadjuvant sintilimab combined with chemotherapy in patients with locally advanced esophageal squamous cell carcinoma. Front Immunol.

[29] Yang Y, Liu J, Liu Z, Zhu L, Chen H, Yu B, Zhang R, Shao J, Zhang M, Li C, Li Z. Two-year outcomes of clinical N2-3 esophageal squamous cell carcinoma after neoadjuvant chemotherapy and immunotherapy from the phase 2 NICE study. J Thorac Cardiovasc Surg. 2023;S0022-5223(23)00782-1. 10.1016/j.jtcvs.2023.08.056 37696429

[30] Shun Lu. Perioperative toripalimab + platinum-doublet chemotherapy vs chemotherapy in resectable stage II/III non-small cell lung cancer (NSCLC): Interim event-free survival (EFS) analysis of the phase III Neotorch study. ASCO Plenary Series (April). Abstract 425126.

[31] Wakelee H, Liberman M, Kato T, Tsuboi M, Lee SH, Gao S, Chen KN, Dooms C, Majem M, Eigendorff E (2023). Perioperative Pembrolizumab for Early-Stage non–small-cell Lung Cancer. N Engl J Med.

[32] Xi M, Yang Y, Zhang L, Yang H, Merrell KW, Hallemeier CL, Shen RK, Haddock MG, Hofstetter WL, Maru DM (2019). Multi-institutional analysis of recurrence and Survival after Neoadjuvant Chemoradiotherapy of Esophageal Cancer: impact of histology on recurrence patterns and outcomes. Ann Surg.

[33] Guo X, Wang Z, Yang H, Mao T, Chen Y, Zhu C, Yu Z, Han Y, Mao W, Xiang J (2023). Impact of Lymph Node Dissection on Survival after Neoadjuvant Chemoradiotherapy for locally advanced esophageal squamous cell carcinoma: from the results of NEOCRTEC5010, a Randomized Multicenter Study. Ann Surg.

